# Root Associated *Bacillus* sp. Improves Growth, Yield and Zinc Translocation for Basmati Rice (*Oryza sativa*) Varieties

**DOI:** 10.3389/fmicb.2015.01286

**Published:** 2015-11-18

**Authors:** Muhammad Shakeel, Afroz Rais, Muhammad Nadeem Hassan, Fauzia Yusuf Hafeez

**Affiliations:** Department of Biosciences, COMSATS Institute of Information TechnologyIslamabad, Pakistan

**Keywords:** bio fertilizer, PGPR, rhizobacteria, zinc, rice, super basmati, basmati-385, 16S rRNA gene

## Abstract

Plant associated rhizobacteria prevailing in different agro-ecosystems exhibit multiple traits which could be utilized in various aspect of sustainable agriculture. Two hundred thirty four isolates were obtained from the roots of basmati-385 and basmati super rice varieties growing in clay loam and saline soil at different locations of Punjab (Pakistan). Out of 234 isolates, 27 were able to solubilize zinc (Zn) from different Zn ores like zinc phosphate [Zn_3_ (PO_4_)_2_], zinc carbonate (ZnCO_3_) and zinc oxide (ZnO). The strain SH-10 with maximum Zn solubilization zone of 24 mm on Zn_3_ (PO_4_)_2_ore and strain SH-17 with maximum Zn solubilization zone of 14–15 mm on ZnO and ZnCO_3_ores were selected for further studies. These two strains solubilized phosphorous (P) and potassium (K) *in vitro* with a solubilization zone of 38–46 mm and 47–55 mm respectively. The strains also suppressed economically important rice pathogens *Pyricularia oryzae* and *Fusarium moniliforme* by 22–29% and produced various biocontrol determinants *in vitro*. The strains enhanced Zn translocation toward grains and increased yield of basmati-385 and super basmati rice varieties by 22–49% and 18–47% respectively. The Zn solubilizing strains were identified as *Bacillus* sp. and *Bacillus cereus* by 16S rRNA gene analysis.

## Introduction

Rice is an important cereal crop growing across the world and is a major staple food of Asian population. In current scenario of intensive farming, soils are continuously depleting in macro and micro nutrients especially in wheat-rice cropping system (Rana et al., 2015). Among micro nutrients, Zn is a highly essential nutrient required throughout the life cycle of rice (Holler et al., 2014).

Farmers use chemical fertilizers to make up the deficiency of essential nutrients and thus achieve high yields. Irrational use of chemical fertilizers has led to severe environmental problems especially contamination of underground water due to leaching and pollution of atmosphere through gaseous emissions (Lockhart et al., 2013; Wu and Ma, 2015). Moreover, fluctuations in price and non-availability of chemical fertilizers due to energy crisis and other reasons pose a major constraint in sustainable crop production. Under these circumstances, plant growth promoting rhizobacteria (PGPR) may offer a valuable alternative to chemical fertilizers.

This is because PGPR live freely in soil, colonize plant roots aggressively and establish symbiotic association with plants. Existence of PGPR with the plant roots is generally classified by two environments *viz*; rhizosphere and endosphere. Rhizosphere represents the soil volume under the direct influence of root while endosphere represents the internal tissue of root (Timm et al., 2015). The strains inhabiting rhizosphere and endosphere are called rhizobacteria and endophytes respectively.

PGPR enrich soil with major plant nutrients such as nitrogen (N) by fixing it from the atmosphere, phosphorous (P), and potassium (K) by solubilizing them from the soil (Patel et al., 2015; Pii et al., 2015; Zahid et al., 2015). They also assist in bioavailability of Zn by solubilizing it from various ores like Zn_3_ (PO_4_)_2_, ZnCO_3_, and ZnO (Abaid-Ullah et al., 2015; Sirohi et al., 2015). In addition to provide macro and micronutrients to the plants, PGPR also protect them from pathogens. They suppress the activity of pathogens by producing numerous antifungal metabolites like siderophores, hydrolytic enzymes, and antibiotics (Chowdhury et al., 2015). Therefore, they could be utilized as an alternative to the chemical fertilizers and fungicides and hence may ensure sustainable agriculture production, environmental safety and lower production cost.

This argument is supported by earlier reports where inoculation of plants with PGPR has resulted in the improved nutrition, vigorous plant growth and high yield (El-Sayed et al., 2014; Majeed et al., 2015). Many effective strains have been formulated as biofertilizers. The use of registered biofertilizers and microbial technologies has become a widely accepted strategy in the current intensive agricultural practices prevailing throughout the world (Shen et al., 2015). The PGPR strains identified so far belong to genus *Pseudomonas, Ochrobacterum, Bacillus, Azosperillum, Azotobacter, Rhizobium, Stenotrophomonas, Serratia*, and *Enterobacteria* (Hassan et al., 2010; Ma et al., 2011; Abaid-Ullah et al., 2015). Highly positive effects of these species on the growth of various crops such as sugarcane, maize, wheat, rice, canola, sunflower and other vegetables have been observed both *in vitro* and *in vivo* under variable climatic conditions (Hassan et al., 2011; El-Sayed et al., 2014). For example, *Kosakonia radicincitans* increased dry weight and N content of yerba mate by 183 and 30% (Bergottini et al., 2015). Wheat yield was improved by 9% upon inoculation with a consortium of *Bacillus thuringiensis* and *Serratia* sp. (Abaid-Ullah et al., 2015; Pereg and McMillan, 2015).

In addition to an increase in yield, PGPR also significantly affect the nutrients uptake by plants. This property of rhizobacteria has drawn the attention of researchers to exploit them in cereals bio fortification. Ramesh et al. (2014) reported that Zn solubilizing strains of *Bacillus aryabhattai* improved Zn mobilization in wheat and soybean. Recent studies conducted at our laboratory (Abaid-Ullah et al., 2015) reveiled that certain strains of *Serratia* sp., *Pseudomonas* sp., and *Bacillus* sp., enhanced Zn translocation toward wheat grains by 7–12% compared to that of chemical Zn (Abaid-Ullah et al., 2015).

The plant–microbe interaction is quite complex phenomena and effect of this interaction on growth and physiological processes occurring in the life of plant has been explored in detail. It has been widely reported that there is high diversity in origin and function of PGPR and their growth promoting potential may be highly specific to certain soils, plant species, genotypes and cultivars (Lucy et al., 2004; Mehta et al., 2015; Zahid et al., 2015). Hence, a thorough investigation of native bacterial communities, their population and characteristics is required to assess the diversity of indigenous bacteria and their distribution in the rhizoplane of certain crops (Bulgarelli et al., 2013; Piromyou et al., 2013).

Effects of PGPR strains vary for different crops growing in various soil types under variable climatic conditions. Therefore, it is necessary to cultivate region-specific microbial strains for the development of suitable bio inoculum to obtain maximum yield and nutrient content of a specific crop (Farag et al., 2013; Habibi et al., 2014). In view of these facts, present study was designed to isolate indigenous bacterial strains from the rice endosphere growing on clay loam and saline soil at different locations. These strains were screened *in vitro* for Zn solubilization potential from different Zn ores. The potent strains were characterized for other plant growth promoting traits and inoculated to rice varieties, basmati 385 and super basmati to assess their potential to enhance yield and translocate Zn under net house conditions. The potent strains were identified by 16S rRNA gene analysis.

## Materials and methods

### Sample collection and isolation of bacteria

Representative plants of five rice varieties viz; super basmati, basmati-385, shaheen basmati, kainat basmati, and basmati-515 growing in two types of soil clay loam and saline were sampled from different locations of Punjab. Three to four fields located in the radius of one kilometer growing in same soil type were identified per location. Four-five plants of variable vigor were selected from each field, uprooted with bulk rhizospheric soil, and pooled up to make a representative sample. The samples were placed individually in paper bags, labeled and transported to lab. Bacterial endophytes were then isolated by following the method of Surette et al. (2003) with certain modifications. Briefly, roots of each plant were separated and thoroughly washed with tap water to remove any adhering soil. The root tissues of each sample were mixed thoroughly and then surface disinfected by a 3 min treatment with commercial bleach (5.25% available chlorine), transferred to a 3% hydrogen peroxide solution for 3 min and finally rinsed three times with sterile milli-Q water followed by air drying in sterile filter paper under the safety cabinet. One gram of the roots was crushed and grinded in sterilized mortar and pestle. The potentially endophytic bacteria were isolated by serial dilution plating of sterilized crushed root on Luria Bertani (LB) agar plates (Hassan et al., 2010). LB agar plates were incubated at 28 ± 2°C for 24–36 h. The individual colonies appearing on the plates were picked and purified by re streaking on LB agar plates. The purified strains were preserved in 20% glycerol at −80°C.

### Screening of Zn solubilizing bacteria

Potential of isolates to solubilize Zn from various ores such as zinc sulfide (ZnS), ZnO, Zn_3_(PO_4_)_2_, and ZnCO_3_ was tested on Bunt and Rovira agar medium (Bunt and Rovira, 1955). The bacterial strains were grown in LB overnight. Five microliter of each bacterial suspension having optical density (OD) normalized to 0.5 was inoculated on specific plates containing 0.1% of the respective ore. The inoculated plates were incubated at 30°C for 36–96 h. Appearance of halo zone around the colonies indicated their potential to solubilize Zn which was estimated by measuring the zone diameter. There were three biological replicates and the experiment was repeated twice.

### Morphological and biochemical characterization of potent Zn solubilizing strains

Two strains exhibiting maximum potential to solubilize Zn from various ores were selected for morphological and biochemical characterization. For each test, the strains were freshly grown in LB broth overnight and normalized to the OD-600 of 0.5 before inoculation on respective plate.

### Gram reaction and antibiotic resistance

The Zn solubilizing strains were characterized for Gram reaction and intrinsic resistance to antibiotics following the method of Vincent (1970). Briefly, the strains were inoculated on agar plates and spreaded by swab. Antibiotic discs of levofloxacin (5 μg), streptomycin (10 μg), piperociline (100 μg), amoxyciline (10 μg), tetracycline (30 μg), kanamycine (30 μg), vanlomycine (30 μg), and minocycline (30 μg) were placed on the plate of each strain and incubated at 28 ± 2°C for 24–48 h. Inhibition of bacterial growth was observed around each antibiotic disc and strains were designated as highly resistant, moderate resistant, moderate susceptible and highly susceptible on the basis of inhibition zone diameter as recommended by the manufacturer.

### Phosphorus (P) and potassium (K) solubilizing activity

Ability of strains to solubilize the major plant nutrients (P, K) were tested on Pikovskaya agar containing tricalcium phosphate as insoluble phosphate source and Aleksandrov agar having potassium aluminum silicate as source of insoluble inorganic potassium respectively (Pikovskaya, 1948; Kumar et al., 2012). Each bacterial culture was spot inoculated in the center of respective agar plates. The plates were incubated at 28 ± 2°C for 7–10 days and observed for the appearance of halo zone around the colonies. Size of the zone diameter around the colonies provided a semi quantitative potential of P and K solubilization of the strains. The experiment was repeated twice with three biological replicates.

### Determination of indole-3-acetic acid and siderophores

Indole-3-acetic acid (IAA) production by the Zn solubilizers was qualitatively determined by growing the strains on LB agar plates supplemented with 100 μg mL^−1^ of tryptophan (Shrivastava and Kumar, 2011). A 0.5 cm deep cavity of 1–2 cm diameter was made by sterile cork borer. A 100 μL of freshly grown culture was inoculated in each cavity. The inoculated plates were incubated at 28 ± 2°C. After 16 h incubation, the bacterial colonies were removed from the cavities with the help of sterile cotton swab and approximately 200 μL of IAA reagent consisting of (1 mL of 0.5 M FeCl_3_ mixed in 50 mL of 35% HClO_4_) was added in the cavity and observed for change in color. Appearance of pink halo zone around the cavity indicated production of IAA by the bacterial culture. Qualitative production of siderophores by the bacterial strains was detected on the Chrome-azurol S (CAS) medium (Schwyn and Neilands, 1987). Each bacterial culture was inoculated separately on CAS agar plates and incubated at 28 ± 2°C for 72 h. The plates were observed for the change in color i.e., orange to yellow and zone diameter was measured to estimate the production of siderophores semi quantitatively.

### Antagonism against economically important pathogens of rice

Two economically important pathogens of rice, *Pyricularia oryzae* causing rice blast and *Fusarium moniliforme* causing bakanae disease of rice were used as test strains to study the antagonistic potential of potent Zn solubilizers. The antagonism was tested by dual culture assay as described by Spence et al. (2014) with certain modifications. The fungal disc of diameter 6 mm was placed at the center of potato dextrose agar (PDA) petri plate. The bacterial culture was spotted at a distance of 4 cm from fungal disc. LB broth instead of bacterial culture was used in mocked (control). The PDA plates were sealed with parafilm and incubated at 28 ± 2°C for 8–10 days. The mycelial diameter of fungus growing out from the edge of bacterial colony was measured. Percentage inhibition of fungal mycelium was calculated by using the following formula:

%inhibition=[(C–T)×100)/C]

Where C = mycelium diameter (cm) of the fungus growing in the control plate and T = mycelium diameter (cm) of the fungus growing in the bacterial treated plates. The experiment was repeated twice with three biological replicates each time.

### Determination of HCN (Hydrogen Cyanide)

Production of HCN was determined by following the method of Miller and Higgins (1970) with certain modifications. The bacterial cells were inoculated on LB agar plates amended with 4.4 g glycine L^−1^. A piece of filter paper having diameter 6 cm was dipped in solution consisting of 0.5% picric acid, 1% Na_2_CO_3_ and placed in the upper lid of each petri plates. The plates were wrapped with parafilm and incubated at 28 ± 2°C for 48–72 h. The change in color of filter paper from yellow to brown was used as indicator for HCN production.

### Determination of hydrolytic enzymes

Hydrolytic enzymes production such as protease, cellulase, and glucanase were detected on the agar plates containing skim milk, carboxy methylcellulase and laminarin respectively (Hassan et al., 2011; Kumar et al., 2012; Abraham et al., 2013). The bacterial strains were inoculated on the respective agar plates and incubated at 28 ± 2°C for 4–7days. Development of halo zone around the colonies indicated enzyme production. Zone of exo β-1, 3-glucanase was observed after staining with congo red (Nagpure et al., 2014).

### Molecular identification of Zn solubilizing bacteria

Zn solubilizing bacteria were identified at molecular level by sequencing 16S rRNA gene. The genomic DNA of bacterial strains was extracted by CTAB extraction (Wilson, 1987). A 1500 bp 16S rRNA gene was amplified by using the primers P1 (5′-AGAGTTTGATCCTGGTCAGAACGAACGCT-3′) and P6 (TACGGCTACCTTGTTACGACTTCACCCC - 3′) as described by Tan et al. (1997). PCR reaction mixture consisting of 10–15 ng DNA, 1.5 mM MgCl_2_,1 X PCR buffer, 200 μM of each, dATP, dCTP, dGTP, and dTTP (Fermentas), 10 mM of each primer, and 1.0–1.5 U of Taq polymerase (Fermentas) was amplified in thermocycler (Peq lab Germany) with the amplifying conditions; initial denaturation at 95°C for 5 min, 25 cycles (94°C for 1 min, 56°C for 1 min, 72°C for 1.75 min) followed by final extension at 72°C for 5 min (Tan et al., 1997; Hassan et al., 2010). The amplified 16S rRNA gene was analyzed on 1% agarose gel and compared with 1 kb DNA ladder (Fermentas). Specific band of 16S rRNA gene was eluted from the gel and purified by using the Gel Extraction Kit (Qiagen). The purified PCR product was sequenced commercially by Macrogen Inc. (Korea). The 16S rRNA gene sequence was annotated, analyzed on BLAST and identified on the basis of closest homologous strain.

### Effect of Zn solubilizing bacteria on rice plants

Effect of Zn solubilizing strains inoculation on plant growth, yield and grain Zn concentration on two rice varieties basmati 385 and basmati super was examined in cleaned earthen pots (20 cm × 30 cm) under net house conditions. The pots were filled with 5 Kg sterilized clay loam soil. NPK fertilizer was applied at the rate of 40, 30, and 20 mg kg^−1^ of soil in the form of urea, single super phosphate and potassium sulfate respectively. Phosphorous (P) and K were applied in single dose before sowing the plants while N was applied in three split doses. The experiment was laid out in a completely randomized design (CRD) with three replications per treatment. Zinc sulfate (ZnSO_4_) at the rate of 7.5 mg kg ^−1^ of soil was used as Zn in respective treatments. There were eight treatments *viz*. T1 = un-inoculated plants (Negative control), T2 = Zn (positive control), T3 = Zn solubilizing strains SH-10, T4 = Zn solubilizing strains SH-17, T5 = Consortium of Zn solubilizing strains SH-10 and SH-17, T6 = Zn solubilizing strains SH-10 + Zn, T7 = Zn solubilizing strains SH-17 + Zn, T8 = Consortium of Zn solubilizing strains + Zn.

Roots of 30 days old rice plants obtained from nursery were surface sterilized and transferred aseptically to the pots. The Zn solubilizing rhizobacteria were inoculated as soil drenching near the plant roots after 2 days of seedling transplant. The bacterial strains were grown in LB broth in a 250 mL Erlenmeyer flask on a shaking incubator at 100 rev min^−1^, 28 ± 2°C for overnight. The cells were pelleted by centrifugation and dissolved in 0.85% saline with a cell OD = 0.45 (~10^9^ CFU mL^−1^). One mL of this cell suspension was applied near the root of each seedling. Sterile saline without bacteria was applied in negative control. The pots were kept in net house during the months of July to October (Natural season of crop). The plants were irrigated when needed until maturity. A second dose of bio inoculants was applied after 45 days of 1st inoculation. The plants were harvested during the month of October and observed for all agronomic traits like number of tillers, plant height, panicle length, thousand grain weight and yield except the leaf chlorophyll content which was measured at anthesis stage. Three leaves per plant were randomly selected and chlorophyll content of each leaf was measured by SPAD meter (Minolta, Tokyo, Japan) from different places (Ranganathan et al., 2006). Plant height was measured from ground level to the tip of panicle by using a measuring rod. The number of tillers were counted by uprooting each plant. The plants of each pot were threshed to separate grains and straw which were weighed separately. Average values of respective parameters were computed and expressed per pot.

### Determination of grain Zn content and Zn translocation index (ZTI)

Plants of four treatments viz un-inoculated plants (T1), Zn (T2), consortium of strains (T5) and consortium of strains along with Zn (T8) were selected for determining the Zn content in shoot and grains. The Zn analysis was carried-out commercially by the Nuclear Institute for food and Agriculture (NIFA), Peshawar, KPK, Pakistan. Zn translocation index (ZTI) toward rice grains was calculated by using the formula (Rengel and Graham, 1996).

ZTI=[Zn concentration in grains/Zn concentration in shoot]×100

### Statistical analysis

The data were subjected to analysis of variance using statistical package Genstat 9.2 (VSN International Ltd., Hemel Hempstead, Hertfordshire, UK, Abaid-Ullah et al., 2015). The differences among various treatment means were compared using the Fisher's protected least significant differences test (LSD) at probability level (*P* ≤ 0.05) (Steel and Torrie, 1980).

## Results

### Prevalence of Zn solubilizing bacteria in rice endosphere

**Figure 1:**
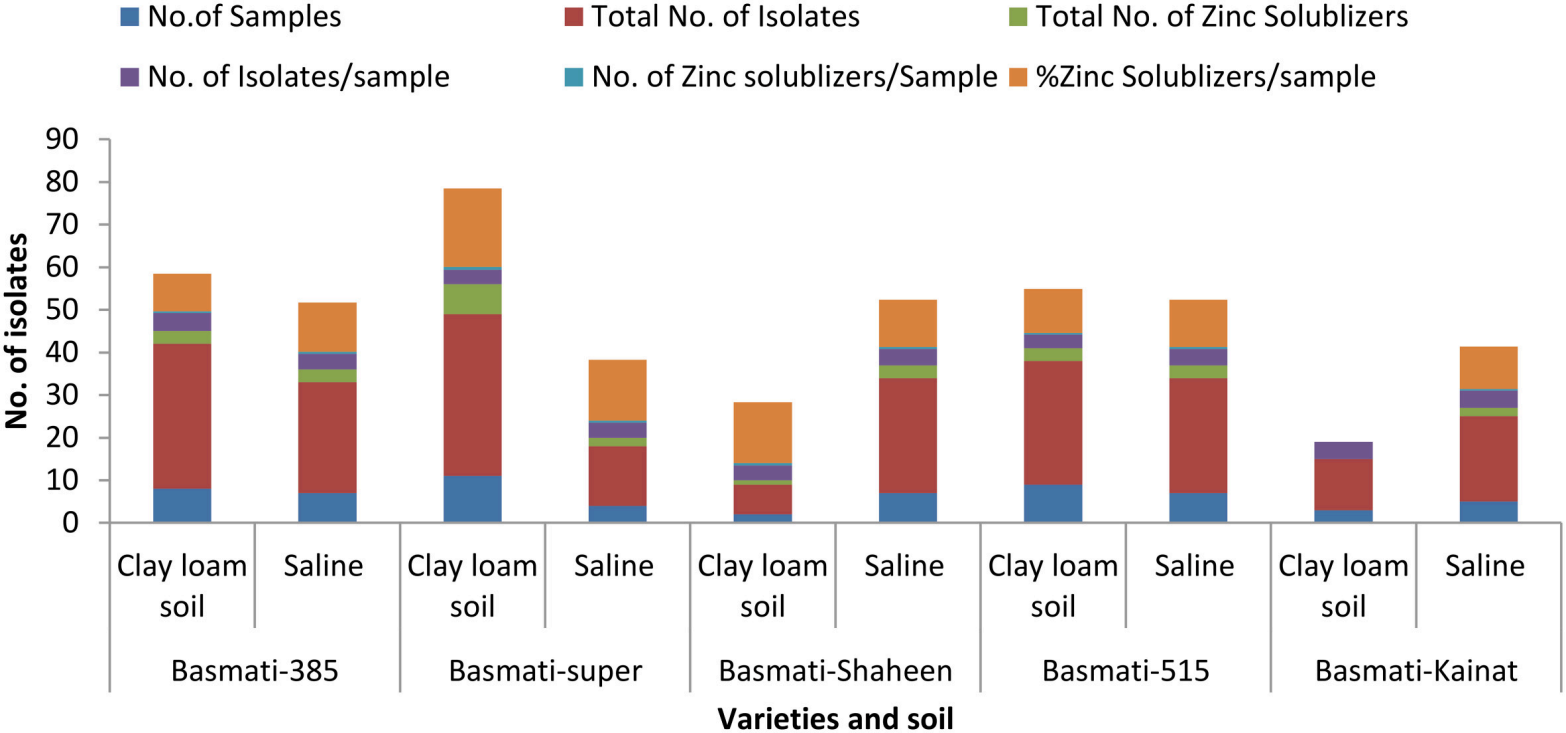
**Prevalence of zinc solubilizing bacteria in the endosphere of different rice varieties**.

**Figure 2:**
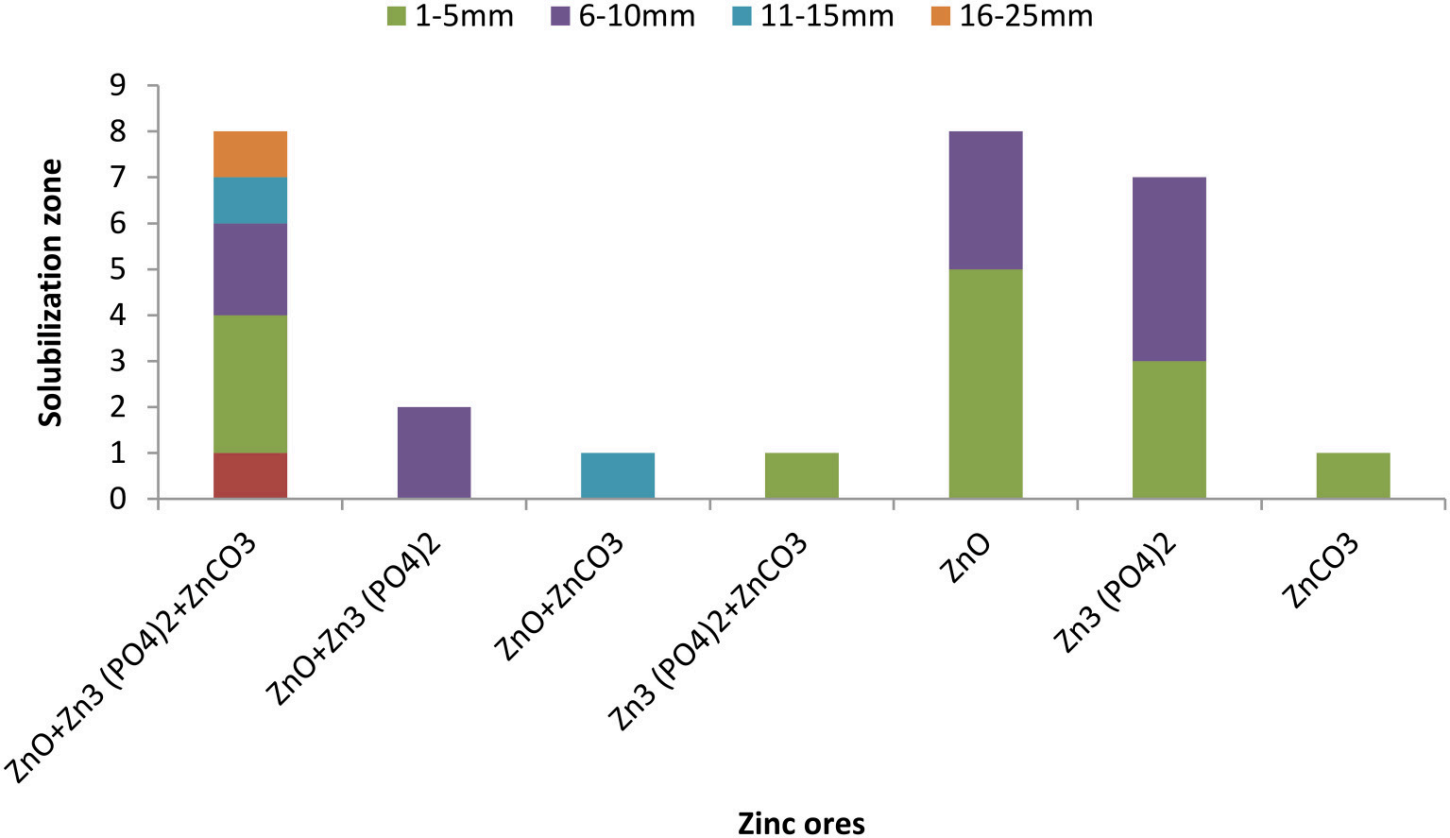
**Number of isolates capable to solubilize various zinc ores with different solubilization zone**.

A total of 234 isolates were obtained from different rice varieties growing at varying locations. Number of isolates in rice endosphere were found to be variable i.e., 3–4 isolates per sample (Figure 1). Twenty seven isolates solubilized Zn either from one or more Zn ores with a solubilization zone of 1–24 mm (Figure 2, Table S1). Distribution of Zn solubilizers associated with rice endosphere was highly variable i.e., 0–18% per sample depending upon the variety and soil conditions (Figure 1). Two strains SH-10 and SH-17 showing maximum solubilizing zone on respective Zn ores were designated as potent Zn solubilizers and selected for further studies.

### Morphological and plant growth promoting (PGP) traits of the potent Zn solubilizers

The Zn solubilizers were Gram positive rod. The strain SH-10 resisted all the antibiotics except levofloxacin (5 μg), tetracycline (30 μg), vanlomycine (30 μg), and minocycline (30 μg) while strain SH-17 showed resistance against all the antibiotics. The strains solubilized P and K with a solubilization zone of 38–46 mm and 47–55 mm respectively but did not produce IAA and HCN. They also inhibited the growth of *P. oryzae* and *F. moniliforme* by 22–30% and produced the antifungal metabolites protease, cellulase and glucanase. The strain SH-10 did not produce siderophores (Table 1).

**Table 1:** **Plant growth promoting traits of Zn solubilizing bacteria isolated from the rice varieties endosphere**.

**Traits**	**Strains**	
Nutrient solubilization^*^	***Bacillus* sp. SH-10**	***Bacillus cereus* SH-17**
Phosphorous	38.0^b^	46.0^a^
Potassium	47.0^b^	55.0^a^
ANTAGONISM^**^		
*P. oryzea*	23.7^b^	30.0^a^
*F. moniliforme*	22.0^a^	22.3^a^
ANTIFUNGAL METABOLITES^***^		
Glucanase	42.2^a^	141.1^a^
Protease	10.9^b^	16.9^a^
Cellulase	15.2^b^	74.5^a^
Siderphore	0.0^b^	53.7^a^

Values are mean of three replicates and bearing different letters in the same row are significantly different from each other according to the analysis of variance (p < 0.05).

*, *** The values are solubilization zones in mm.

** The values are percent inhibition of fungal mycelium.

### Effect of Zn solubilizers on yield and yield components of rice varieties

#### Basmati-385

Zn solubilizing strains significantly increased the yield and yield components of rice variety basmati-385 (Table 2). Maximum effect on yield (45.8 g/pot) and yield components i.e., plant height (97.1 cm), number of tillers (31.4/pot), panicle length (20.6 cm), chlorophyll content (34.5) was observed in co-inoculation of the Zn solubilizers along with the Zn followed by the Zn treatment (Table 2). The strain SH-17 and Zn resulted a 35.4 g grain yield/pot which was statistically at par with that of consortium of Zn solubilizers and Zn (45.8 g grain yield/pot) and only Zn treatment (34.5 g grain yield/pot).

**Table 2:** **Effect of zinc solubilizing bacteria on yield and yield components of rice variety basmati -385 under net house conditions**.

**Treatments**	**Plant height (cm)**	**No. Tiller/Pot**	**Chlorophyll content**	**Panicle Length(cm)**	**Grain Yield/Pot (g)**	**Straw weight/Pot (g)**	**Biological Yield/pot (g)**	**Percent increase over control**	
								**Grain yield**	**Biological yield**
Control	52.7^e^	18.7^c^	13.7^e^	7.4^d^	23.3^c^	48.9^d^	72.2^e^	–	–
Zinc	88.2^b^	22.4^bc^	30.7^b^	18.2^ab^	34.5^b^	68.1^bc^	102.6^bc^	32.5	29.6
*Bacillus* sp. SH-10	69.2^c^	20.8^bc^	20.2^d^	12.3^c^	32.8^bc^	55.8^d^	88.5^d^	29.0	18.4
*B. cereus* SH-17	61.1^d^	19.4^c^	19.1^d^	8.7^d^	30.0^bc^	55.5^d^	85.5^de^	22.0	15.5
Consortium of *Bacillus* sp. SH-10 and *B. cereus* SH-17	71.1^c^	21.8^bc^	24.7^c^	17.2^b^	33.2^bc^	59.3^cd^	92.6^cd^	30.0	22.0
*Bacillus* sp. SH-10 + Zinc	92.5^ab^	25.8^b^	32.5^ab^	20.4^a^	32.0^bc^	76.0^ab^	108.0^b^	27.2	33.1
*B. cereus* SH-17 + Zinc	92.1^ab^	24.8^b^	31.3^b^	18.8^ab^	35.4^ab^	68.5^bc^	103.9^bc^	34.2	30.5
Consortium of *Bacillus* sp. SH-10 and *B. cereus* SH-17 + Zinc	97.1^a^	31.4^a^	34.5^a^	20.6^a^	45.8^a^	83.9^a^	129.7^a^	49.1	44.3
*P*- value	43.5	6.3	80.9	27.7	3.1	11.8	16.9	–	
*F*-value	< 0.001	0.002	< 0.001	< 0.001	0.034	< 0.001	< 0.001		

Control, non-inoculated; Zinc, Zinc sulfate (ZnSO_4_) at the rate of 7.5 mg kg^−1^ of soil. Values are mean of three replicates and bearing different letters in the same column are significantly different from each other according to the analysis of variance (p < 0.05).

#### Super basmati

Effect of Zn solubilizing bacteria on the yield and yield components of rice variety super basmati was significant (Table 3). Highest grain yield/pot (38.6 g) and yield components i.e., plant height (100.9 cm), tillers/pot (33.8), chlorophyll content (34.9) and 1000 grain weight (23.2 g) were observed in super basmati plants treated with the consortium of strains and Zn followed by that of treated with strain SH-10 and Zn with grain yield/pot (32.3 g), plant height (92.3 cm), tillers/pot (30.6), chlorophyll content (32.9), and 1000 grain weight (21.4 g). Moreover, effect of treatments i.e., strain SH-10 and Zn, SH-17 and Zn and only Zn on basmati rice was statistically same but different from that of other treatments (Table 3).

**Table 3:** **Effect of zinc solubilizing bacteria on yield and yield components of rice variety super basmati under net house conditions**.

**Treatments**	**Plant Height (cm)**	**No. Tiller/Pot**	**Chlorophyll Content**	Panicle Length^*^(cm)	**1000 Grain Weight (g)**	**Grain Yield/pot (g)**	**Straw weight/pot (g)**	**Bio Yield/pot(g)**	**Percent increase over control**	
									**Grain yield**	**Biological yield**
Control	66.8^e^	18.2^d^	20.1^e^	20.1	12.2^f^	20.3^d^	41.0^f^	61.6^f^	–	–
Zinc	79.1^c^	28.5^b^	31.7^abc^	25.9	19.1^c^	30.4^b^	57.1^c^	88.2^c^	33.2	30.2
*Bacillus* sp. SH-10	72.5^de^	21.3^d^	27.6^cd^	22.4	15.5^e^	24.8^c^	46.2^e^	71.7^e^	18.1	14.1
*B. cereus* SH-17	71.2^de^	20.1^d^	26.2^d^	21.3	15.1^e^	24.6^c^	46.0^e^	71.6^e^	17.5	14.0
Consortium of *Bacillus* sp. SH-10 and *B. cereus* SH-17	76.7^cd^	25.2^c^	30.0^bcd^	24.7	17.4^d^	26.4^c^	52.2^d^	79.3^d^	23.1	22.3
*Bacillus* sp. SH-10 + Zinc	92.3^b^	30.6^ab^	32.9^ab^	28.2	21.4^b^	32.3^b^	61.0^b^	94.0^b^	37.2	34.5
*B. cereus* SH-17 + Zinc	81.8^c^	29.9^b^	32.2^ab^	26.5	20.2^bc^	31.9^b^	59.1^bc^	91.3^bc^	36.4	32.5
Consortium of *Bacillus* sp. SH-10 and *B. cereus* SH-17 + Zinc	100.9^a^	33.8^a^	34.9^a^	29.7	23.2^a^	38.6^a^	66.1^a^	105.4^a^	47.4	41.6
*P*-value	32.6	27.7	10.8	1.8	71.1	64.3	70.9	145.6	–	
*F*-value	< 0.001	< 0.001	< 0.001	0.172	< 0.001	< 0.001	< 0.001	< 0.001		

Control, non-inoculated; Zinc, Zinc sulfate (ZnSO_4_) at the rate of 7.5mg kg^−1^ of soil. Values are mean of three replicates and bearing different letters in the same column are significantly different from each other according to the analysis of variance (p < 0.05).

* Non-significant.

### Zn translocation index of rice varieties

Effect of Zn solubilizers on the ZTI of both varieties was similar (Table 4). Highest ZTI was observed in the rice plants treated with consortium of strains (ZTI = 1.6–1.7) followed by the plants treated with only Zn (ZTI = 1.3–1.4) or consortium of strains and Zn (ZTI = 1.3–1.4). The lowest ZTI was observed in the un-inoculated plants (ZTI = 0.9–1.1). This clearly shows the role of Zn solubilizers in Zn translocation toward rice grains.

**Table 4:** **Effect of zinc solubilizing bacteria on zinc translocation index in rice varieties basmati-385 and super basmati**.

**Treatment**	**Straw (mg/Kg)**	**Husk (mg/Kg)**	**Grains (mg/Kg)**	**Harvest index**
**BASMATI-385**				
Control	19.0^b^	22.7^d^	17.7^b^	0.9^c^
Zinc	20.3^b^	28.0^c^	27.3^a^	1.3^ab^
Consortium of *Bacillus* sp. SH-10 and *B. cereus* SH-17	19.0^b^	34.3^a^	30.7^a^	1.6^a^
Consortium of *Bacillus* sp. SH-10 and *B. cereus* SH-17 + Zinc	24.0^a^	31.7^b^	30.0^a^	1.3^bc^
*P*-value	0.011	< 0.001	0.002	0.015
**SUPER BASMATI**				
Control	19.7^b^	22.0^b^	22.3^b^	1.1^c^
Zinc	23.0^a^	31.0^a^	31.7^a^	1.4^b^
Consortium of *Bacillus* sp. SH-10 and *B. cereus* SH-17	18.7^b^	30.0^a^	32.3^a^	1.7^a^
Consortium of *Bacillus* sp. SH-10 and *B. cereus* SH-17 + Zinc	22.0^a^	34.0^a^	30.7^a^	1.4^b^
*P*-value	0.006	0.004	< 0.001	0.002

Control, non-inoculated; Zinc, Zinc sulfate (ZnSO_4_) at the rate of 7.5 mg kg^−1^ of soil.

Values are mean of three replicates and bearing different letters in the same column are significantly different from each other according to the analysis of variance (p < 0.05).

### Molecular identification of potent Zn solubilizers

The potent Zn solubilizers were identified as *Bacillus* sp. and *Bacillus cereus* on the basis of 16S rRNA gene analysis. The sequences of strains were submitted to NCBI Gene Bank database under accession numbers KT380823 and KT380824.

## Discussion

PGPR inhabit wide range of crops growing under varying agricultural practices and commonly used as bio inoculants. In addition to their synergistic effect on plant's growth and yield, they have strong potential to enhance the Zn content of cereals (Sharma et al., 2013; Wang et al., 2014; Abaid-Ullah et al., 2015). Utilization of such PGPR to enhance (Zn) content of rice grains could be a promising strategy to minimize the Zn deficiency in human beings. Keeping in view the specific advantages of indigenous strains such as host adaptability and field efficacy, certain potent strains were screened *in vitro* and *in vivo* to enhance the growth, yield and Zn content of rice varieties.

Among the various potentially endophytic isolates associated with rice varieties, a significant difference in the number of Zn solubilizers was observed in different varieties and soil types. Maximum Zn solubilizers were enumerated from the endosphere of variety super basmati growing in clay loam soil while minimum strains were obtained from the endosphere of basmati kainat. However, in saline soil growing plants, prevalence of Zn solubilizers in the endosphere of all rice varieties was almost similar. The significant difference in the number of Zn solubilizers among different varieties and soil types may be due to the fact that plant microbe interaction is highly dependent on soil conditions and plant genotype (Schreiter et al., 2014; Sugiyama and Yazaki, 2014; Belimov et al., 2015). Variation in quantity and composition of microbes associated with the rhizosphere and endosphere of different plants, species and even varieties within same species have already been well documented (Beneduzi et al., 2013; Lagos et al., 2014; Ling et al., 2014). In a recent study, Hameed et al. (2015) has reported that the diversity of bacteria inhabiting rice endosphere and their distribution as well as PGP characteristics were dependent on multiple factors such as host's genotype, soil characteristics and nutrients. The PGPR strains have been screened from the rhizosphere of rice grown in different countries (de Souza et al., 2013) but less attention has been paid to explore the Zn solubilizers associated with rice, a crop which grows in flooded conditions and numerous factors affect the Zn availability in such conditions (Lefèvre et al., 2014; Abaid-Ullah et al., 2015).

In this study, a large number of potentially endophytic isolates were recovered from the rice endosphere but a very few strains depicted Zn solubilization potential. The strains capable to solubilize maximum Zn from respective ores were further tested for their morphological traits like Gram's reaction, cell shape and colony morphology. These traits are essential to recognize specific bacterial strain and tentative identification (Mohamad et al., 2014).

A potent strain exhibiting multiple PGPR traits must be able to resist extreme environmental conditions so that it may survive and maintain optimum population throughout the life cycle of specific crop. Competitive ability of strain to survive in environment is strongly correlated with its intrinsic antibiotic resistance. The Zn solubilizing strains resisted most of the important antibiotics which depicted their ability to tolerate the environmental stress. PGPR especially belonging to genus *Bacillus* sp. are able to survive in adverse environmental conditions due to their ability to form endospores and change the fatty acid patterns depending on variable colonizing niches (Checinska et al., 2015; Diomande et al., 2015). Thus, the Zn solubilizers screened in this study could maintain their population in rice rhizosphere throughout the crop cycle.

Phosphorous (P) and K are the macro nutrients required by rice and the other field crops for growth and optimum yield (Saleque et al., 2013; Wang et al., 2013; Damon et al., 2014; Zorb et al., 2014). Uptake of these macro (N, P, K) and micronutrients (Zn) by the plants from soil is mutually dependent (Bouain et al., 2014). They experience a complex processes in soil and exhibit dynamic equilibrium between insoluble and soluble forms under the influence of soil pH. This equilibrium could be affected by the acid secretion and other activities of soil microbiota, thereby enhancing their availability to plant roots for absorption (Saravanan et al., 2004). Zn application enhanced the uptake of macronutrients (N, P, K) in rice and influenced the biological properties of soil (Pooniya et al., 2012). These facts further advocate the worth of Zn solubilizers screened in this study as they also solubilize the mineral P and K.

Ability of rhizobacteria to solubilize Zn, K, and (PO_4_)^−3^ is dependent on secretion of acids such as gluconic acids, lactic acid, malic acid and oxalic acid etc (Estrada et al., 2013; Zhang and Kong, 2014; Abaid-Ullah et al., 2015). These three elements [Zn, K, and (PO4)^−3^] are released from their insoluble compounds by following the same basic mechanism of acidification and thus correlate the metabolism of each other.

A PGPR could be an ideal candidate for the development of bioformulation provided it possesses multiple characteristics for plant growth promotion. The Zn solubilizers suppress growth of economically important pathogens of rice *P. oryzae* and *F. moniliforme* and produce several secondary metabolites involved in antagonistic activity of the PGPR. These metabolites include siderophores which chelate iron and deprive the pathogen from an important source of nutrition, thereby inhibiting the pathogen by creating a competitive environment (Aznar and Dellagi, 2015). The strains also produce hydrolytic enzymes like glucanase, cellulase and protease which hydrolyze the various components of cell wall, thereby paralyzing the pathogen which ultimately leads to death of the pathogen (Nagpure et al., 2014). Thus, Zn solubilizing rhizobacteria exhibit multiple PGP traits *in vitro* which are similar to the earlier findings (El-Sayed et al., 2014; Abaid-Ullah et al., 2015). Suppression of pathogens by Zn solubilizers augment their potential as an effective bioinoculant because they can protect the plants from devastating diseases along with providing nutrition (Abaid-Ullah et al., 2015). In certain bacteria, the properties of Zn solubilization and pathogen suppression has been found to be interlinked. As reported by Saravanan et al. (2007), presence of solubilized Zn in the culture filtrate enhanced the antagonistic activity of *Gluconacetobacter diazotrophicus*. The siderophores produced by the antagonistic bacteria chelate iron and play major role in their antagonistic activity. On the other hand, Fe ^+3^ oxidation under aerobic soil conditions limits the Zn availability (Gao, 2007). This fact advocates the production of siderophores as a mechanism adopted by PGPR for enhancing Zn availability.

*In vivo* evaluation of the strains exhibiting multiple plant growth promoting properties in laboratory conditions is necessary to develop an effective bio inoculum. Hence, the potent strains were inoculated on two rice varieties, basmati 385 and super basmati grown in net house under the natural growth conditions. The strains inoculated in consortium along with chemical Zn significantly enhanced the yield and related parameters in present research. Effect of PGPR on rice yield has already been reported (Estrada et al., 2013; Ji et al., 2014) but they synergize the effect of chemical Zn on rice basmati varieties, is being firstly reported. As the strains showed their potential to increase yield and yield related traits *in vivo* they may serve as useful bio inoculum.

In addition to increase yield, the Zn solubilizing rhizobacteria also enhanced Zn translocation to the rice grains in similar way as that of chemical Zn. These findings were similar to earlier studies where PGPRs translocated Zn toward rice grains (Tariq et al., 2007; Sharma et al., 2014; Vaid et al., 2014; Wang et al., 2014). The Zn translocation toward rice grains may depend upon the ability of rhizobacteria to enhance Zn availability by executing multiple mechanisms such as mineralization, solubilization and induction of physiological processes in rice involved in Zn uptake just like the induction of systemic resistance in rice against pathogens (Lucas et al., 2014). However, it needs to explore in future studies. There was no significant increase in Zn translocation toward rice grains when consortium of Zn solubilizers was inoculated along with the chemical Zn. This may be due to the inherent Zn uptake potential of the tested rice varieties, i.e., basmati 385 and super basmati. These findings suggest potential use of strains in rice bio fortification with Zn.

The potent Zn solubilizers capable to enhance growth, yield and grain Zn content of rice were identified as *Bacillus* sp. and *Bacillus cereus*. These findings are similar to that of earlier reports in which zinc solubilizers have been found to be recurrent among various bacterial taxa (He et al., 2011; Abaid-Ullah et al., 2015). Certain strains of *B. cereus* are opportunistic human pathogens but numerous strains isolated from plant rhizoplane exhibit PGPR traits and their potential as bio fertilizer is well documented (Niu et al., 2011; Chun Juan et al., 2012; Ramesh et al., 2014; Chowdhury et al., 2015). Thus, the *Bacillus* sp. capable to solubilize nutrients, produce siderophores and antagonize the pathogens by different mechanisms could be used as effective bio inoculants.

## Conclusion

The Zn solubilizing bacteria associated with indigenous host enhance the growth, yield and Zn content by providing nutrition as well as disease protection. Such strains could be ideal candidate to develop bioformulation to improve not only rice yield but also produce Zn fortified rice grains. Moreover, these findings could help researchers to explore the mechanisms involved in PGPR mediated Zn translocation in cereals. The Zn solubilizing strains reported in this study would be made available upon request as per institutional material transfer agreement policy.

### Conflict of interest statement

The authors declare that the research was conducted in the absence of any commercial or financial relationships that could be construed as a potential conflict of interest.
