# Supplementary material for: Root Associated Bacillus sp. Improves Growth, Yield and Zinc Translocation for Basmati Rice (Oryza sativa) Varieties
Source: Front Microbiol. 2015 Nov 18;6:1286. doi: 10.3389/fmicb.2015.01286 (PMC4649038; doi:10.3389/fmicb.2015.01286)
Supplement: Supplementary file 1 [file Table1.DOCX]

**Suppl Table (1): Zinc solubilizing potential of bacteria isolated from the endosphere of rice varieties growing in saline and clay loam soil.**

| **Strain code** | **ZnO** | **Zn_3_ (PO_4_)_2_** | **ZnCO_3_** |
| --- | --- | --- | --- |
| **SH-1** | **19.0 ^a^** | **5.0^gh^** | **12.7 ^b^** |
| **SH-2** | **11.3 ^c^** | **7.0^ef^** | **15.3 ^a^** |
| **SH-3** | **14.7 ^b^** | **9.3 ^cd^** | **14.0 ^ab^** |
| **SH-4** | **10.7 ^c^** | **2.3 ^i^** | **10.0 ^c^** |
| **SH-5** | **8.3 ^d^** | **10.0 ^c^** | **0.0 ^g^** |
| **SH-6** | **0.0 ^i^** | **4.7 ^gh^** | **0.0 ^g^** |
| **SH-7** | **0.0 ^i^** | **7.0 ^ef^** | **0.0 ^g^** |
| **SH-8** | **7.3 ^de^** | **9.0 ^cd^** | **0.0 ^g^** |
| **SH-9** | **8.7 ^d^** | **16.3 ^b^** | **6.3 ^de^** |
| **SH-10** | **5.7 ^efg^** | **24.0 ^a^** | **5.0 ^e^** |
| **SH-11** | **4.3 ^fg^** | **7.0 ^ef^** | **7.0 ^d^** |
| **SH-12** | **0.0 ^i^** | **6.3 ^fg^** | **3.0 ^f^** |
| **SH-13** | **8.0 ^d^** | **0.0 ^j^** | **0.0 ^g^** |
| **SH-14** | **2.3 ^h^** | **0.0 ^j^** | **0.0 ^g^** |
| **SH-15** | **5.3 ^fg^** | **0.0 ^j^** | **0.0 ^g^** |
| **SH-16** | **6.0 ^ef^** | **0.0 ^j^** | **0.0 ^g^** |
| **SH-17** | **14.0 ^b^** | **0.0 ^j^** | **15.0 ^a^** |
| **SH-18** | **4.3^fg^** | **0.0 ^j^** | **0.0 ^g^** |
| **SH-19** | **4.3 ^fg^** | **0.0 ^j^** | **0.0 ^g^** |
| **SH-20** | **4.3 ^fg^** | **0.0 ^j^** | **0.0 ^g^** |
| **SH-21** | **4.0 ^gh^** | **0.0 ^j^** | **0.0 ^g^** |
| **SH-22** | **0.0 ^i^** | **0.0 ^j^** | **2.3 ^f^** |
| **SH-23** | **0.0 ^i^** | **4.3 ^h^** | **0.0 ^g^** |
| **SH-24** | **0.0 ^i^** | **8.3 ^cde^** | **0.0 ^g^** |
| **SH-25** | **0.0 ^i^** | **9.7 ^cd^** | **0.0 ^g^** |
| **SH-26** | **0.0 ^i^** | **2.0 ^i^** | **0.0 ^g^** |
| **SH-27** | **0.0 ^i^** | **8.0^def^** | **0.0 ^g^** |

ZnO = Zinc oxide; Zn_3_ (PO_4_)_2_ = Zinc phosphate; ZnCO_3_ = Zinc carbonate

Values are mean of three replicates and bearing different letters in the same column are significantly different from each other according to the analysis of variance (p< 0.05).
